# Correction: Visible-light photooxidation in water by ^1^O_2_-generating supramolecular hydrogels

**DOI:** 10.1039/d0sc90102d

**Published:** 2020-05-29

**Authors:** Sankarsan Biswas, Mohit Kumar, Andrew M. Levine, Ian Jimenez, Rein V. Ulijn, Adam B. Braunschweig

**Affiliations:** Advanced Science Research Center, Graduate Center, City University of New York 85 St. Nicholas Terrace New York NY 10031 USA abraunschweig@gc.cuny.edu; Department of Chemistry, Hunter College 695 Park Avenue New York NY 10065 USA; PhD Program in Chemistry, Graduate Center, City University of New York New York NY 10016 USA; PhD Program in Biochemistry, Graduate Center, City University of New York New York NY 10016 USA; The High School for Math, Science, and Engineering, The City College of New York 240 Convent Avenue New York NY 10031 USA

## Abstract

Correction for ‘Visible-light photooxidation in water by ^1^O_2_-generating supramolecular hydrogels’ by Sankarsan Biswas *et al.*, *Chem. Sci.*, 2020, **11**, 4239–4245, DOI: 10.1039/C9SC06481H.

We regret that a related reference was omitted from the original article, which is given here as ref. 1. Draper *et al.* previously reported the synthesis of functionalized DPP with phenylalanine side chains. This structure is one of the low molecular weight gelators that we studied as a catalyst in our paper, although the aims of the two manuscripts are very different. Draper *et al.* used this molecule to show triggered hydrogelation by slowly decreasing pH. Finally, they studied electronic properties and alignment of these DPP-Phe fibers in the paper. Although we cited this work in our ESI, this work is relevant to our work and we would like to cite this paper in the main manuscript and acknowledge their work properly. Citations to ref. 1 should be included in the following sentences in the Introduction and Results and discussion sections:

On page 4239 in the sentence beginning “The assembly of the gels…” we would like to include a citation to ref. 1, in addition to ref. 35 in the original paper.

On page 4240 “We have assessed the catalytic activities of three such DPP-based supramolecular hydrogels that vary in their amino acid side chains, where they include either tyrosine (**Y**), phenylalanine (**F**)^1^ or leucine (**L**).”

On page 4240 “First, the DPP core was prepared by Dieckmann condensation of dimethyl succinate and thiophene-2-carbonitrile^1^ with 87% yield.”

On page 4240 “The core was then functionalized at the heterocyclic N with *tert*-butyl acetate *via* an S_N_2 substitution^1^ with 47% yield.”

The Royal Society of Chemistry apologises for these errors and any consequent inconvenience to authors and readers.
